# Single‐Cell Metabolic Imaging and Digital Scoring of Fat Tissue Remodeling by Label‐Free Metabolic Microscopy

**DOI:** 10.1002/advs.202513042

**Published:** 2026-03-02

**Authors:** Myeongseop Kim, Constantin Berger, Alexander Wolf, Alina Peteranderl, Martin Klingenspor, Vasilis Ntziachristos, Yongguo Li, Miguel A. Pleitez

**Affiliations:** ^1^ Institute of Biological and Medical Imaging Bioengineering Center Helmholtz Zentrum München Neuherberg Germany; ^2^ Institute of Pharmacology and Toxicology University Hospital University of Bonn Bonn Germany; ^3^ Chair of Biological Imaging Central Institute for Translational Cancer Research (TranslaTUM) School of Medicine and Health & School of Computation Information and Technology Technical University of Munich Munich Germany; ^4^ Institute of Computational Biology (ICB) Helmholtz Munich Neuherberg Germany; ^5^ Chair for Molecular Nutritional Medicine School of Life Sciences Weihenstephan Technical University of Munich Freising Germany; ^6^ Munich Institute of Robotics and Machine Intelligence (MIRMI) Technical University of Munich Munich Germany

**Keywords:** adipocytes, brown fat, browning, MiROM, optoacoustic microscopy, UCP1

## Abstract

Adipose tissue plasticity and functional heterogeneity play a central role in maintaining energy homeostasis, and their malfunction leads to metabolic disorders such as obesity, diabetes, and cardiometabolic disease. Rapid, single‐cell metabolic imaging of intact fat tissue not only extends our understanding of metabolic dynamics and heterogeneity but also holds great potential as a tool for clinical diagnosis. However, the use of exogenous labels and dyes in conventional optical microscopy results in tissue deformation and requires time‐consuming tissue preparation. Here, we demonstrated single‐cell imaging of metabolic changes and heterogeneity in freshly excised adipose tissues that can distinguish tissue types without the need for exogenous labels using bond‐specific, non‐destructive, mid‐infrared optoacoustic microscopy (MiROM) that allows preserving the native tissue architecture with minimal sample preparation time. Further leveraging MiROM, we monitored intracellular molecular and morphological changes during postnatal remodeling of adipose tissue when metabolic characteristics of adipocytes undergo a transient drastic change. Additionally, we developed a quantitative spatial tissue analysis tool (Q‐SAT) to predict the spatial distribution of white fat‐ and brown fat‐like features, providing a robust digital scoring method for adipose tissue phenotypic assessment. Collectively, we implemented MiROM as an enabling technology to provide fast, label‐free metabolic imaging of unprocessed adipose tissue, opening a new perspective for understanding and characterizing the morpho‐functional dynamics of adipose tissue remodeling.

## Graphical abstract

Mid‐infrared optoacoustic microscopy (MiROM) acquires lipid‐ and protein‐ associated vibrational contrast in intact fat tissue without dyes, preserving native tissue architecture. Through lateral and axial segmentation, MiROM tracks intrinsic intracellular changes during postnatal remodeling. A quantitative spatial analysis tool (Q‐SAT) maps white‐ and brown‐like features, enabling rapid digital phenotyping of adipose tissue.

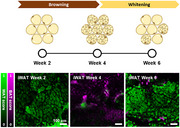

## 1 Introduction

Adipose tissue is an extraordinarily plastic and heterogeneous organ in mammals that controls energy balance, glucose, and lipid metabolism [1, 2, 3, 4, 5, 6, 7, 8] and is commonly classified into two types: white adipose tissue (WAT), which stores energy, and brown adipose tissue (BAT), which dissipates energy and contributes to thermogenesis and metabolic control. The remodeling capacity of adipose tissue not only allows the body to adapt rapidly to variations in energy supply and demand but also plays a major role in orchestrating metabolic health [1]. The remarkable plasticity of adipose tissue is further exemplified by the ability of adipocytes to interconvert between white and brown phenotypes. Brown‐like adipocytes or brite (brown‐in‐white, or also beige, or recruitable brown) adipocytes share the capacity for uncoupling protein 1 (UCP1)‐mediated non‐shivering thermogenesis yet present a discrete cell type. These beige adipocytes can be found interspersed within WAT, demonstrating the complexity of fat depots and the need for understanding not only their plasticity but also heterogeneity [9, 10]. As impaired plasticity and loss of heterogeneity lead to diminished or abnormal responses to physiological challenges and thus drive the progression of metabolic diseases, a comprehensive understanding of adipose tissue plasticity and heterogeneity is crucial for developing new potential therapeutic targets for managing obesity and other metabolic disorders, ultimately advancing relevant clinical applications.

Metabolic imaging enables the acquisition of spatio‐temporal information on biological responses to external stimuli and can provide insight into disease progression and therapeutic response. However, many metabolic imaging modalities rely on exogenous labels or markers, which may have limited biodistribution, perturb biological function, or are not readily available. Moreover, metabolic responses are often highly dynamic and heterogeneous, underscoring the need to interrogate metabolic plasticity with high sensitivity and ideally at the single‐cell level. In parallel, recent advances in single‐cell and spatial multi‐omics have enabled increasingly comprehensive characterization of cellular heterogeneity, including integrated transcriptome‐proteome measurements extending toward spatially resolved readouts [11]. At the same time, label‐free molecular fingerprinting approaches at the single‐cell level have progressed, including SERS‐based spatial fingerprinting strategies for probing intracellular biochemical states in live cells [12]. Single‐cell metabolomics is emerging as a direct method for quantifying metabolic heterogeneity at the single‐cell level [13]. However, many platforms still rely on substantial sample processing (e.g., tissue dissociation) and specialized workflows, which complicates rapid assessment of intact tissue architecture. More broadly, “metabolic fingerprinting” frameworks have been explored for rapid, label‐free histopathology and disease stratification [14]. Despite these advances, conventional adipose tissue assays still frequently rely on bulk measurements that obscure cell‐to‐cell variability, and transcriptome‐based approaches often infer metabolic state indirectly rather than measuring biochemical content in intact tissue. Together, this motivates technologies that preserve native tissue architecture while enabling label‐free, spatially resolved metabolic phenotyping with minimal processing time.

Label‐free chemical imaging techniques, unlike imaging with exogenous labels, enable the analysis of morphological characteristics and molecular information of tissue while preserving the native architecture of the tissue of interest and avoiding the time‐consuming sample preparation steps typically required for exogenous staining. In particular, vibrational spectroscopic methods based on Raman scattering [15, 16] or mid‐infrared (mid‐IR) absorption [17, 18] have been developed for label‐free chemical imaging. Raman‐scattering microscopy modalities, i.e., spontaneous Raman scattering, coherent anti‐Stokes Raman scattering (CARS) and stimulated Raman scattering (SRS) microscopy, have been applied in cell and tissue studies to characterize and to monitor the various biomolecules (e.g., lipid and protein) during metabolic activities [16, 19]. For instance, Raman‐scattering microscopy has recently revealed distinct cellular phenotypes of white and brown adipocytes during adipogenic differentiation [20, 21]. However, the low Raman scattering cross‐sections of biomolecules—typically on the level of 10^−30^ cm^2^—result in low detection sensitivity (low mm range) and high irradiation energies (100's of mW) on the studied specimens, which can lead to sample perturbation by photodamage [22]. On the contrary, mid‐IR absorption cross‐sections are up to eight orders of magnitude larger than Raman‐scattering [23, 24, 25], and thus mid‐IR spectroscopy and imaging offer the potential to significantly improve sensitivity and specificity for label‐free analytical histology in the mid‐IR region (4000–400 cm^−1^) [26]. However, as conventional mid‐IR spectroscopy relies primarily on optical detection, the applicability of mid‐IR microscopy on living cells and unprocessed tissues is limited by the significant IR absorption of water and signal loss caused by negative‐contrast optical detection, i.e., the detected signal becomes weak as optical absorption increases [27, 28]. As a result, assessment of excised tissues by optically‐detected mid‐IR microscopy requires time‐consuming tissue preparation steps similar to those required in conventional histology, i.e., tissue sectioning slices <10 µm thickness [27].

Unlike conventional vibrational microscopy, mid‐IR optoacoustic microscopy (MiROM) uses detection of optically generated acoustic signals—i.e., optoacoustic (OA) signals—which are less attenuated and scattered than purely optical signals. MiROM enables obtaining mid‐IR absorption contrast from thick, unprocessed, freshly‐excised tissues (up to 6 mm thickness) with minimal preparation steps [29]—thus preserving the integrity of unprocessed tissue during histological analysis while providing high chemical sensitivity. MiROM has been applied to the histological characterization of carotid atherosclerosis [30], which visualized high‐risk plaque features with molecular assignment. MiROM has also been applied for label‐free differentiation between inflamed vs. non‐inflamed WAT [31] based on spectroscopic feature selection, achieving a considerable reduction of total imaging time (by a 15x factor) as compared to laser scanning confocal microscopy, which uses labels for identifying structures associated with inflammation [31]. Furthermore, MiROM has demonstrated depth‐selective analysis of OA signal applied to minimize the interference of superficial skin layers to enhance the sensitivity for non‐invasive glucose sensing [32].

Here, we took advantage of the high heterogeneity and plasticity of adipose tissue as a model system and hypothesized that the depth‐selectivity and label‐free chemical specificity imaging features of MiROM make it possible to identify the intrinsic hallmarks of WAT and BAT in fresh unprocessed fat tissues. We found that, with minimal sample preparation, MiROM allowed us to identify intrinsic biochemical characteristics that can distinguish BAT from WAT and monitor the transient intracellular behavior of white fat depots observed during the postnatal development of mice. Specifically, in this work, for the first time, we characterized the spatial‐temporal distribution of lipid and protein changes in beige adipocytes at the cellular level during postnatal tissue remodeling, i.e., browning and whitening. Overall, MiROM combined with Q‐SAT provides a robust biotechnology for label‐free histological assessments of adipose tissue, allowing characterization and aiding in understanding the morpho‐functional dynamics of adipose tissue remodeling. Additionally, based on the ability to distinguish spectral characteristics between WAT and BAT, we combine MiROM with a quantitative spatial analysis tool (Q‐SAT) to obtain digital scores of adipocyte phenotypes (WAT&BAT scoring). Q‐SAT operates on MiROM intensities at a set of 10 selected wavenumbers, reducing the required spectral sampling compared to full hyperspectral MiROM across the entire wavenumber range of QCL and thus lowering the data acquisition time to enable fast digital scoring. For comparison, for the same imaging acquisition parameters (1 × 1 mm^2^ FOV at step size of 5 µm), full hyperspectral acquisition (502 wavenumbers) would take 67 h while a 10‐wavenumber acquisition requires only 80 min.

## 2 Results

### 2.1 Intracellular Imaging and Multi‐Spectral Analysis of Adipocytes in Freshly Excised Fat Tissue

**FIGURE 1:**
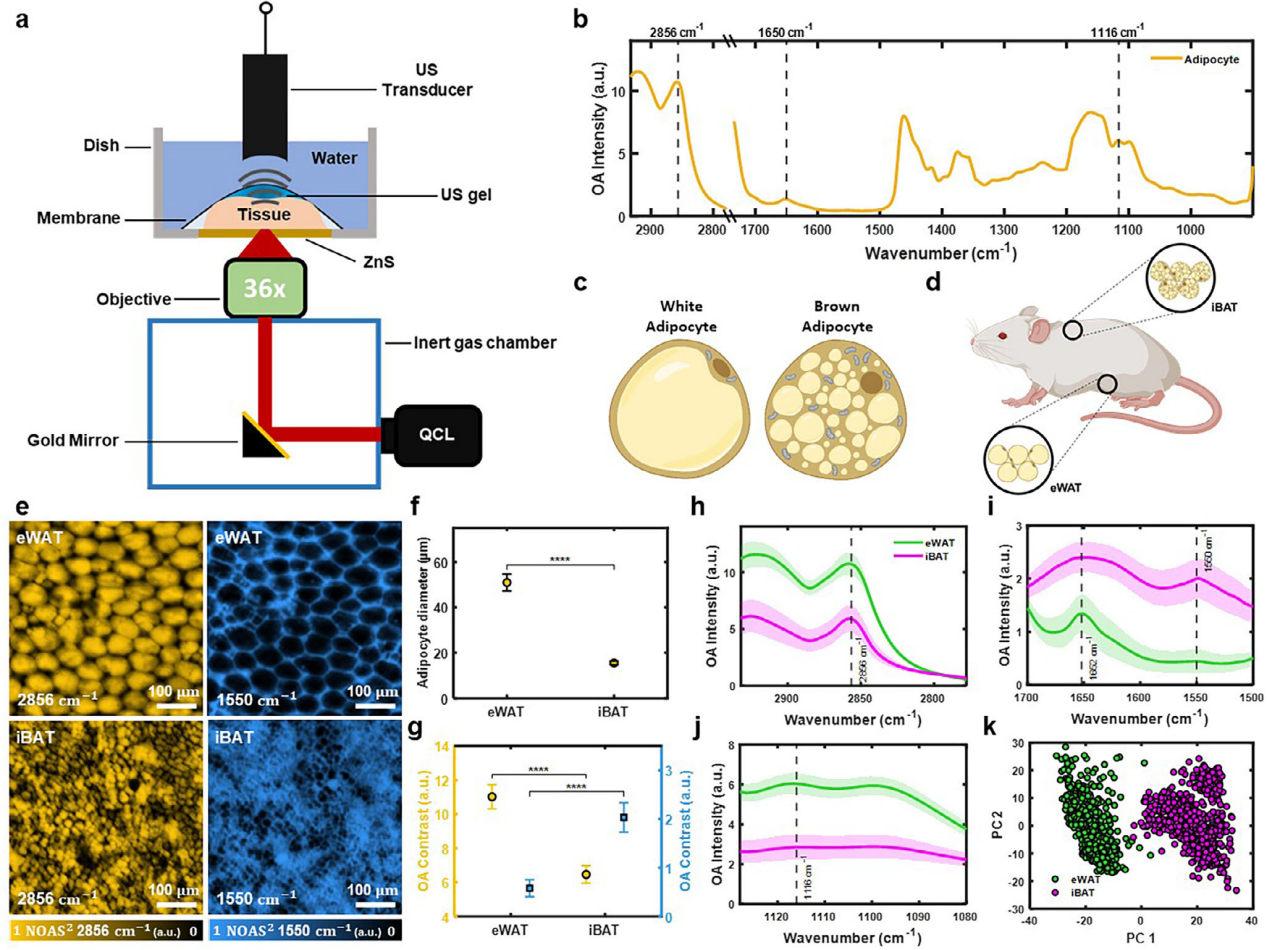
Determination of spectral and morphological hallmarks of white and brown adipose tissue. (a) Schematic representation of mid‐infrared optoacoustic microscopy (MiROM) for label‐free analytical histology of adipose tissue. Ultrasound (US), quantum cascade laser (QCL). (b) Representative optoacoustic (OA) spectra (2932–909 cm^−1^) from a selected adipocyte in adipose tissue. (c) Illustration of a white adipocyte (left) and a brown adipocyte (right). (d) Schematic diagram of adipocyte tissue locations on FVB/NJ mice. The locations of epididymal white adipose tissue (eWAT) and interscapular brown adipose tissue (iBAT) are marked. (e) Depth‐selective OA micrographs—Field‐Of‐View (FOV) of 1  ×  1 mm^2^—of eWAT and iBAT for 2856 cm^−1^ (yellow) and 1550 cm^−1^ (cyan). Normalized optoacoustic signal (NOAS). (f) Comparison of the mean diameter of adipocytes in eWAT and iBAT. The error bars indicate standard deviation (SD). (g) Comparison of digitally isolated adipocyte OA contrast for 2856 and 1550 cm^−1^ micrographs obtained from eWAT and iBAT adipocytes. The error bars indicate SD. (h–j) Depth‐selective OA spectra of eWAT and iBAT adipocytes for three different spectral ranges (h: 2932–2778 cm^−1^, i: 1700–1500 cm^−1^, and j: 1128–1080 cm^−1^). The mean spectra (solid line) and their SD (shaded area) are shown for eWAT and iBAT. (k) Principal component (PC) analysis 2D scatter plot derived from eWAT and iBAT spectra. Results in f, g show statistical analysis for two‐group tissue comparisons (eWAT and iBAT), unpaired two‐sample *t*‐tests (see Methods: Statistical Analysis), of adipocyte sizes and OA contrast per mouse (*n* = 7 mice) for each tissue type. Results in h–k were obtained from 771 eWAT adipocytes and 745 iBAT adipocytes – 7 mice per tissue type. Illustrations c,d were created with BioRender.com.

MiROM is a label‐free chemical imaging technique that preserves the native architecture and intrinsic biomolecular composition of freshly excised tissues and thus enables circumventing the limitations of conventional methods for histological assays (e.g., H&E staining, immunohistochemical staining). To demonstrate MiROM's capabilities for histological assays, we implemented it for label‐free chemical analysis of freshly excised adipose tissue as illustrated in Figure 1a; details on MiROM operation are given in **Methods** and elsewhere [29, 30, 31, 32]. In short, in MiROM, an ultrasound (US) signal is generated by the optical absorption of a diffraction‐limited mid‐IR excitation beam (∼5 µm focus size at 2850 cm^−1^ excitation) illuminating the tissue and detected by a focused ultrasound transducer. The focused mid‐IR excitation beam—from a broadly tunable quantum cascade laser (QCL)—and a focused US detector are confocally aligned to the adipose tissue that is placed on a custom‐designed Petri dish with deionized water for acoustic coupling between the tissue and the US detector. In this configuration, mid‐IR spectra on selected structures of interest in the tissue can be obtained by tuning the excitation wavelength of the QCL across a broad spectral range (2932–909 cm^−1^). Figure 1b shows a representative mid‐IR OA spectrum obtained from white adipocytes. As expected, since lipid droplets in adipocytes are primarily composed of triglycerides [33], the spectrum in Figure 1b is dominated by the spectral features of triglycerides, including symmetric CH_2_ stretching (2856 cm^−1^), C═C stretching (1650 cm^−1^), and C─O stretching vibration of the C─OH group (1116 cm^−1^) [26, 34]. The band around 1650 cm^−1^ is dominated by the amide I band of proteins, with a possible minor contribution from C═C stretching of unsaturated lipids, whereas the band at ∼1550 cm^−1^ corresponds to the amide II band of proteins. Next, a MiROM micrograph is obtained by point‐by‐point raster scanning the sample across the focal plane while acquiring the OA signal at a selected mid‐IR excitation wavelength (Figure S1a,b) and plotting the maximum amplitude projection (MAP) of the OA signal for each measured point. Visualization of the different intrinsic bio‐molecular content of tissues is obtained by acquiring micrographs at multiple vibrational transitions (mid‐IR excitation wavelengths). For instance, as shown in Figure S1a, b, lipid and protein content of epididymal white adipose tissue (eWAT) and interscapular brown adipose tissue (iBAT) are visualized at 2856 and 1550 cm^−1^, respectively. Here, we observe that, although an overlap from different molecular content is expected, the contrast obtained for micrographs at 2856 cm^−1^ is greatly dominated by the lipid content in adipocytes, i.e., the triglycerides in lipid droplets, while the contrast obtained at 1550 cm^−1^ mainly originates from the protein and water content in the extracellular matrix (ECM) with only a small contribution from the interior of adipocytes. As a result, since the ECM is composed of lower lipid and higher protein content compared to adipocytes and vice versa, we observed a considerable contrast difference between adipocytes and extracellular protein structure obtained at 2856 and 1550 cm^−1^, and thus micrographs at these wavelengths appear to be complementary, see Figure S1a,b [35, 36, 37]. At the location of adipocytes, due to adipocytes’ low protein and water content, micrographs at 1550 cm^−1^ appear dark compared to the ECM. However, although rather low, the intracellular protein and water contrast at 1550 cm^−1^ is still expected from adipocytes, but we hypothesized that it is overshadowed by the strong contrast of the surrounding ECM and thus not visible at first sight in the micrographs. This hypothesis was tested and proven positive by closer inspection of the OA signal from these seemingly dark areas, where we found non‐negligible contrast content at the adipocytes’ location, see Figure S1g,h.

Having demonstrated non‐negligible contrast in adipocytes at 1550 cm^−1^ and in order to minimize the overshadowing effect of the surrounding ECM to precisely assess the intracellular content of adipocytes, we performed a digital isolation protocol of adipocyte contrast in two steps. First, we leveraged the depth‐selective abilities of MiROM to reach the inner content of adipocytes by analyzing OA signals at selected depths. Depth‐selective mid‐IR spectral analysis is possible because the time‐resolved (transient) OA signal contains depth information as reported by the arrival time of the ultrasound signal at the transducer—namely, signals from deeper tissue layers arrive with a time delay compared to signals from shallow depths. In this way, depth‐selective information can be retrieved (axial isolation) by time gating the OA transient signals, see Methods, and Figure S1c–f for details. Second, we applied an intensity threshold to the micrographs at 2850 cm^−1^ to generate a mask for 2D localization of the adipocyte‐containing areas (lateral isolation) [32].

In the following, we analyzed the intrinsic intracellular content of adipocytes in adipose tissues by axial and lateral isolation.

### 2.2 Determination of the Morphological and Molecular Hallmarks of WAT and BAT by MiROM

The functional differences between WAT and BAT have been closely associated with the morphological differences of the adipocytes composing these two tissue types; for instance, adipocyte size, adipocyte density (count per unit area in mm^2^), and number of mitochondria as depicted in Figure 1c [38, 39]. In mice, epididymal white adipose tissue (eWAT) is representative for classical WAT, while interscapular brown adipose tissue (iBAT) is representative of classical BAT (Figure 1d) [7, 40]. Based on the strong contrast between adipocytes and extracellular protein structure observed at 2856 cm^−1^, we utilized MiROM to characterize morphological differences in adipocytes between eWAT and iBAT. Specifically, we quantified adipocyte size (diameter) and adipocyte density in both tissue types using samples from 14 mice (eWAT: *n* = 7, iBAT: *n* = 7). Representative depth‐selective OA micrographs acquired at 2856 and 1550 cm^−1^ for eWAT and iBAT are shown in Figure 1e. The diameter of adipocytes in eWAT was, on average, ca. 3.1 times larger than those in iBAT (eWAT: 50± 15 µm, iBAT: 16± 6 µm) while the adipocyte density in eWAT was ca. 8.5 times lower than in iBAT (Figure S2e,f). While adipocyte morphology is sufficient to distinguish classical eWAT and iBAT in our murine model, intermediate remodeling states show overlapping size distributions; thus, we complement morphology with MiROM's bond‐specific contrast to capture biochemical changes during browning and re‐whitening.

Moreover, by digital isolation of adipocyte contrast—i.e., depth‐selected and laterally segmented contrast—we were able to compare eWAT and iBAT adipocyte‐specific molecular content at 2856 and 1550 cm^−1^ (see Figure 1g). Here we found that, at 2856 cm^−1^ the contrast in eWAT is ca. 1.7 times higher than in iBAT while at 1550 cm^−1^ the contrast of iBAT is ca. 3.4 times higher than eWAT. Thus, the vibrational contrast information indicates a lipid‐rich and protein‐poor content for eWAT and a lipid‐poor and protein‐rich content in iBAT. These remarkable morphological and contrast differences between eWAT and iBAT observed at 2856 cm^−1^ (lipid) and 1550 cm^−1^ (protein) represent unique tissue‐specific hallmarks that we used for tissue identification.

Beyond morphology, MiROM was also able to reveal remarkable spectral differences between eWAT‐ and iBAT‐adipocytes in the 2932–909 cm^−1^ spectral range, see Figure 1h–j (resulting spectra from 771 eWAT adipocytes and 745 iBAT adipocytes – 7 mice per tissue type). In particular, spectral differences between eWAT and iBAT were detected at the CH_2_ vibration region (2932–2778 cm^−1^; Figure 1h), the amide I and II bands (amide I band: 1700–1600 cm^−1^, amide II band: 1600–1500 cm^−1^; Figure 1i), and the CO vibration region (1128–1080 cm^−1^; Figure 1j). While the CH_2_ vibrational region (2932–2778 cm^−1^; Figure 1h) and the CO vibrational region (1128–1080 cm^−1^; Figure 1j) are relevant for lipid characterization, the amide I and II bands (1700–1500 cm^−1^) are critical to identifying protein content. From the CH_2_ and the CO vibrational regions—as reported by the area under the curve (AUC), see Methods—we observed ca. two times higher OA signal for eWAT‐adipocytes compared to the OA spectra of iBAT‐adipocytes. Spectral intensity differences in these two regions support the hypothesis that eWAT‐adipocytes have a higher lipid content than iBAT‐adipocytes. From the amide I and II bands, we obtained ca. 2.8 times higher AUC of mean spectra for iBAT‐adipocytes than for eWAT‐adipocytes suggesting that iBAT‐adipocytes contain more protein than eWAT‐adipocytes—most likely, due to the elevated mitochondrial count in iBAT‐adipocytes as compared to eWAT‐adipocytes. In particular, from the spectral region between 1700 and 1500 cm^−1^, we observed the most remarkable spectral differences between eWAT‐ and iBAT‐adipocytes. In iBAT, we observed the two typical absorption bands characteristic of protein content at 1650 and 1550 cm^−1^ (amide I and II, respectively), while for eWAT, we observed only a narrow absorption band at 1650 cm^−1^ (no band at 1550 cm^−1^) with a slope toward 1700 cm^−1^ mostly associated with lipid content. Since the 1550 cm^−1^ band corresponds to the amide II band of proteins, its pronounced presence in iBAT is consistent with the larger fraction of protein‐rich cytoplasm and mitochondria (including UCP1‐rich mitochondrial membranes) relative to lipid volume in brown adipocytes. In contrast, unilocular eWAT adipocytes are dominated by a large triglyceride droplet, which reduces the relative contribution of intracellular proteins within the focal volume and therefore yields a much weaker amid II band absorption. The spectral differences between eWAT‐ and iBAT‐adipocytes (resulting spectra from 771 eWAT adipocytes and 745 iBAT adipocytes – 7 mice per tissue type) are visualized in Figure 1k where a 2D scatter plot obtained by principal component analysis (PCA) shows two clearly defined clusters grouped by spectra similarity, each group representing eWAT‐ or iBAT‐adipocytes. Thus, MiROM is able to morphologically and spectrally distinguish between brown and white adipose tissues.

### 2.3 Label‐Free Longitudinal Monitoring of Postnatal Adipose Tissue Remodeling

**FIGURE 2:**
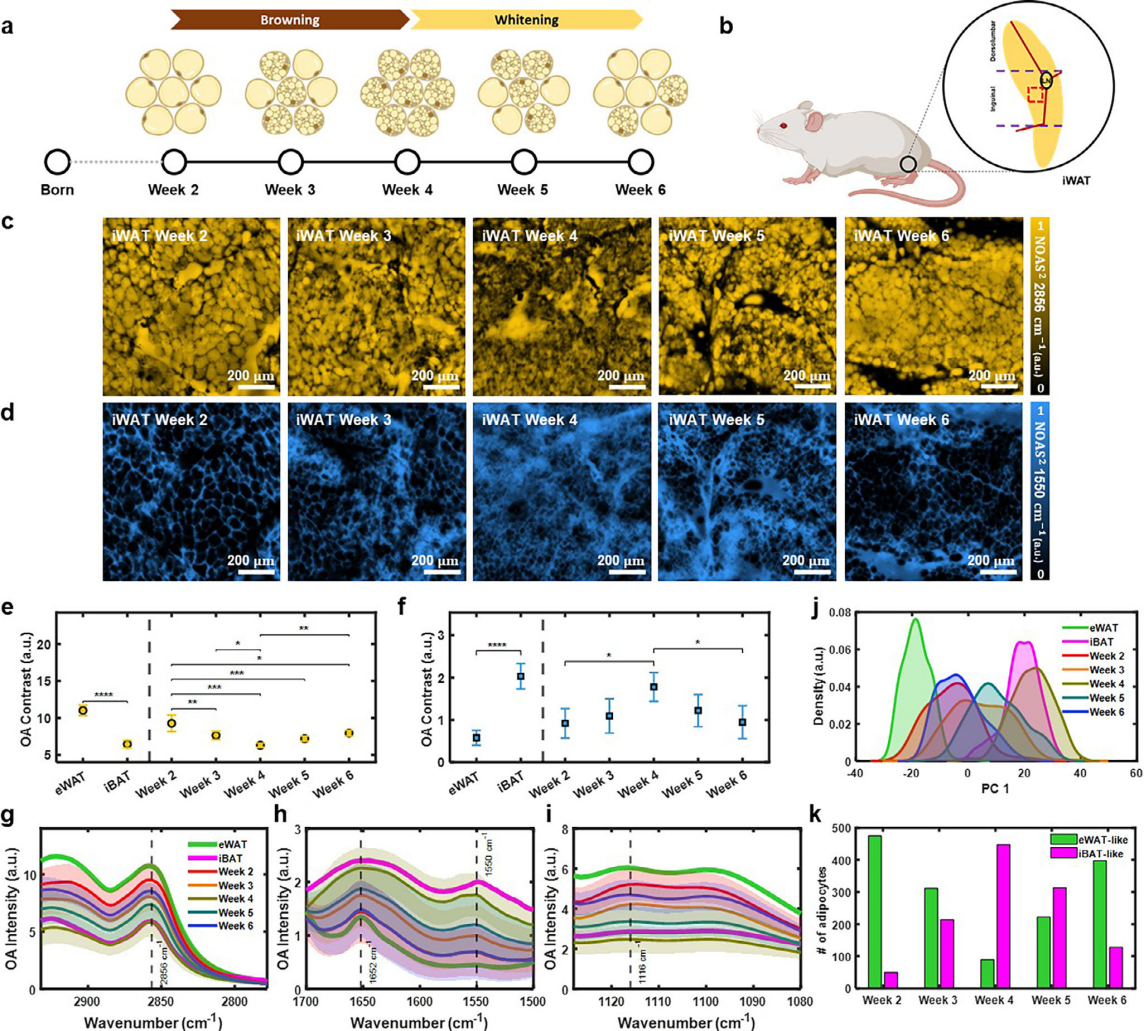
Monitoring morphological and spectral dynamics during postnatal development in inguinal white adipose tissue. (a) Timeline of iWAT transition from postnatal week 2 through week 6 indicating a browning phase during postnatal weeks 2–4 and a subsequent whitening phase from postnatal weeks 4–6. (b) Schematic diagram of inguinal white adipose tissue (iWAT) location on FVB/NJ mice. The region of interest for iWAT, located beneath the lymph node (LN), is marked by a red dashed box. (c,d) Representative example of depth‐selective OA micrographs of iWAT at postnatal weeks 2–6 at 2856 cm^−1^ (c) and 1550 cm^−1^ (d) for a FOV of 1  × 1 mm^2^. (e,f) Mean OA contrast (at 2856 and 1550 cm^−1^) of digitally isolated adipocyte regions in eWAT and iBAT compared to iWAT across postnatal weeks 2–6. The error bars indicate SD. (g–i) Change of depth‐selective OA spectra of iWAT adipocytes from postnatal weeks 2–6 for three different spectral ranges (g: 2932–2778 cm^−1^, h: 1700–1500 cm^−1^, and i: 1128–1080 cm^−1^). The mean spectra (solid line) and their SD (shaded area) are shown for eWAT and iBAT 771 eWAT adipocytes and 745 iBAT adipocytes – 7 mice per tissue type), and iWAT (525 adipocytes of Week 2, 525 adipocytes of Week 3, 537 adipocytes of Week 4, 535 adipocytes of Week 5, and 525 adipocytes of Week 6 – 5 mice per postnatal week). (j) Kernel density estimation of PC1 derived from adipocyte spectra for eWAT, iBAT, and iWAT (postnatal weeks 2–6). (k) Bar chart indicating the estimated amount of eWAT‐like and iBAT‐like adipocytes in iWAT during postnatal weeks 2–6 obtained by a logistic regression model trained on spectra from eWAT and iBAT adipocytes. Results in e,f show statistical analysis for two‐group tissue comparisons (eWAT and iBAT), unpaired two‐sample *t*‐tests, and for comparisons across postnatal weeks, one‐way ANOVA with Bonferroni‐corrected post‐hoc tests, of OA contrast per mouse (eWAT and iBAT – *n* = 7 mice; iWAT across postnatal weeks – *n* = 5 mice) for each tissue type (see Methods: Statistical Analysis). Illustrations a,b were created with BioRender.com.

Besides classical white and brown adipocytes, brown‐like adipocytes (brown‐in‐white, or also beige, or recruitable brown) occurring within WAT under certain conditions (e.g., cold exposure, beta‐adrenergic stimulation, PPARγ agonist treatment) represent a distinct type of thermogenic fat cell and hold great promise for promoting metabolic health [1, 7, 10]. Remarkably, during the first two months of postnatal development, iWAT of mice undergoes a drastic, but transient, remodeling process between a white‐to‐brown (browning – postnatal weeks 2–4) and brown‐to‐white phenotype (whitening – postnatal weeks 4–6) with unclear molecular control and physiological function (Figure S3) [3, 7, 40, 41]. We leveraged this dynamic postnatal adipose tissue remodeling in iWAT as an excellent model to test the robustness of MiROM in monitoring intracellular molecular and morphological changes of browning and whitening, longitudinally. Based on the morphological and molecular hallmarks of WAT and BAT identified using MiROM, we performed longitudinal monitoring of adipose tissue remodeling during postnatal development of mice (Figure 2a). Figure 2b shows the locations where tissues were excised as well as the region of interest (ROI) beneath the lymph node in iWAT where significant beige adipocyte development occurs. As expected, from OA micrographs (Figure 2c,d), we observed a decrease in the size of adipocytes in iWAT during postnatal weeks 2–4 and an increase in the size of adipocytes in iWAT during postnatal weeks 4–6 (Figure S2d,e). In contrast, adipocyte count per unit area exhibited the opposite trend (Figure S2f). The observed changes indicate adipocyte hyperplasia from weeks 2 to 4 and hypertrophy from weeks 4 to 6. These morphological changes were confirmed by hematoxylin and eosin (H&E) staining of the tissues (Figure S3).

For a thorough comparison, vibrational contrast of eWAT, iBAT, and iWAT (eWAT and iBAT: *n* = 7; iWAT at postnatal weeks 2–6: *n* = 5 each) at two channels (lipid: 2856 cm^−1^; protein: 1550 cm^−1^) is shown in Figure 2e,f. The contrast at 2856 cm^−1^ decreases in iWAT from postnatal weeks 2 to 4 and increases again from postnatal weeks 4 to 6, while the contrast at 1550 cm^−1^ follows opposite trends. These observations match the transient browning (postnatal weeks 2–4) and subsequent re‐whitening (postnatal weeks 4–6) phases of iWAT, as confirmed by western blot and whole‐mount immunofluorescence staining (Figure S3). The inverse coupling between lipid contrast (2856 cm^−1^) and protein contrast (1550 cm^−1^) is consistent with transient enrichment of protein‐rich, mitochondria‐dense adipocyte states during browning, followed by a relative loss of mitochondrial or protein content during re‐whitening.

Figure 2g–i shows depth‐selective OA spectra of iWAT‐adipocytes (resulting spectrum from 525 adipocytes of Week 2, 525 adipocytes of Week 3, 537 adipocytes of Week 4, 535 adipocytes of Week 5, and 525 adipocytes of Week 6 – 5 mice per postnatal week) from three different spectral ranges. From the CH_2_ vibration region (2932–2778 cm^−1^) and the CO vibration region (1128–1080 cm^−1^), dynamic spectral changes were observed in iWAT‐adipocytes at postnatal weeks 2–4 (AUC of the mean spectra decreases) and weeks 4–6 (AUC of the mean spectra increases). From the amide I and II bands (1700–1500 cm^−1^), an opposite direction of dynamic spectral change was observed compared to the other two regions. Additionally, we also observed how spectral features change between postnatal weeks 2 and 4, reflecting a transition from eWAT‐like to iBAT‐like spectra. In contrast, changes between postnatal weeks 4 and 6 reflect a transition in the opposite direction, i.e., from iBAT‐like to eWAT‐like spectra. To better understand the spectral dynamics for iWAT during postnatal weeks 2–6 in comparison to eWAT and iBAT, in Figure 2j, we show the kernel density estimation plot of PC1 where a clear spectral transition following tissue browning and tissue whitening is observed.

In order to quantify the iWAT transitions during postnatal weeks 2–6, we developed a metric based on logistic regression trained using depth‐selective OA spectra of adipocytes from eWAT and iBAT (see Methods). In this way, we generated WAT‐ and BAT‐scores for each adipocyte to indicate the similarity of spectra features to those from eWAT and iBAT in terms of probability. Based on the WAT‐ and BAT‐scores, we analyzed iWAT‐adipocytes during postnatal weeks 2–6 in terms of their similarity to eWAT and iBAT, see Figure 2k. Thereby, we observed that the number of iWAT‐adipocytes which is more similar to eWAT decreases from postnatal weeks 2 to 4 and reversely increases from postnatal weeks 4 to 6, while adipocytes spectrally more similar to iBAT increase from postnatal weeks 2 to 4 and decrease from postnatal weeks 4 to 6. Figure 2k represents the trajectory of tissue browning (postnatal weeks 2–4) and the re‐whitening (postnatal weeks 4–6) of iWAT‐adipocytes based on WAT and BAT scores. Hence, adipose tissue remodeling can be analyzed based on the spectral features of adipocytes contained in iWAT by comparison with eWAT‐ and iBAT‐adipocytes. Thus, depth‐selective OA spectral analysis with spectral scoring can be used to detect and quantify the browning (postnatal weeks 2–4) and the re‐whitening (postnatal weeks 4–6) phase of iWAT during postnatal development.

### 2.4 Digital Scoring of Tissue Based on MiROM and Machine Learning Approach

Since the WAT and BAT scores were derived based on the spectra acquired from selected structures of interest, the analysis potentially suffers from a selection bias of manually chosen tissue locations, which could be corrected by performing hyperspectral analysis on the tissues. However, due to the acquisition time required for hyperspectral imaging using MiROM (e.g., 1 × 1 mm^2^ at a step size of 5 µm for 502 wavenumbers will require ∼67 h), the analysis was restricted to a localized spectral analysis of the chosen structures of interest. Nevertheless, to avoid selection bias and obtain spatial information (cell‐to‐cell variations) on adipose tissue remodeling, we extended our tissue scoring method and developed a quantitative spatial analysis tool (Q‐SAT) for adipose tissue. Q‐SAT allows to digitally scoreing adipocyte features based on hyperspectral imaging with a reduced number of wavelengths compared to full spectral analysis to lower the data acquisition time (e.g., 1 × 1 mm^2^ (step size of 5 µm) requires only 80 min over 10 wavenumbers). In that manner, Q‐SAT enabled the unbiased assessment of the spatial distribution of adipose tissue remodeling at the whole tissue level.

**FIGURE 3:**
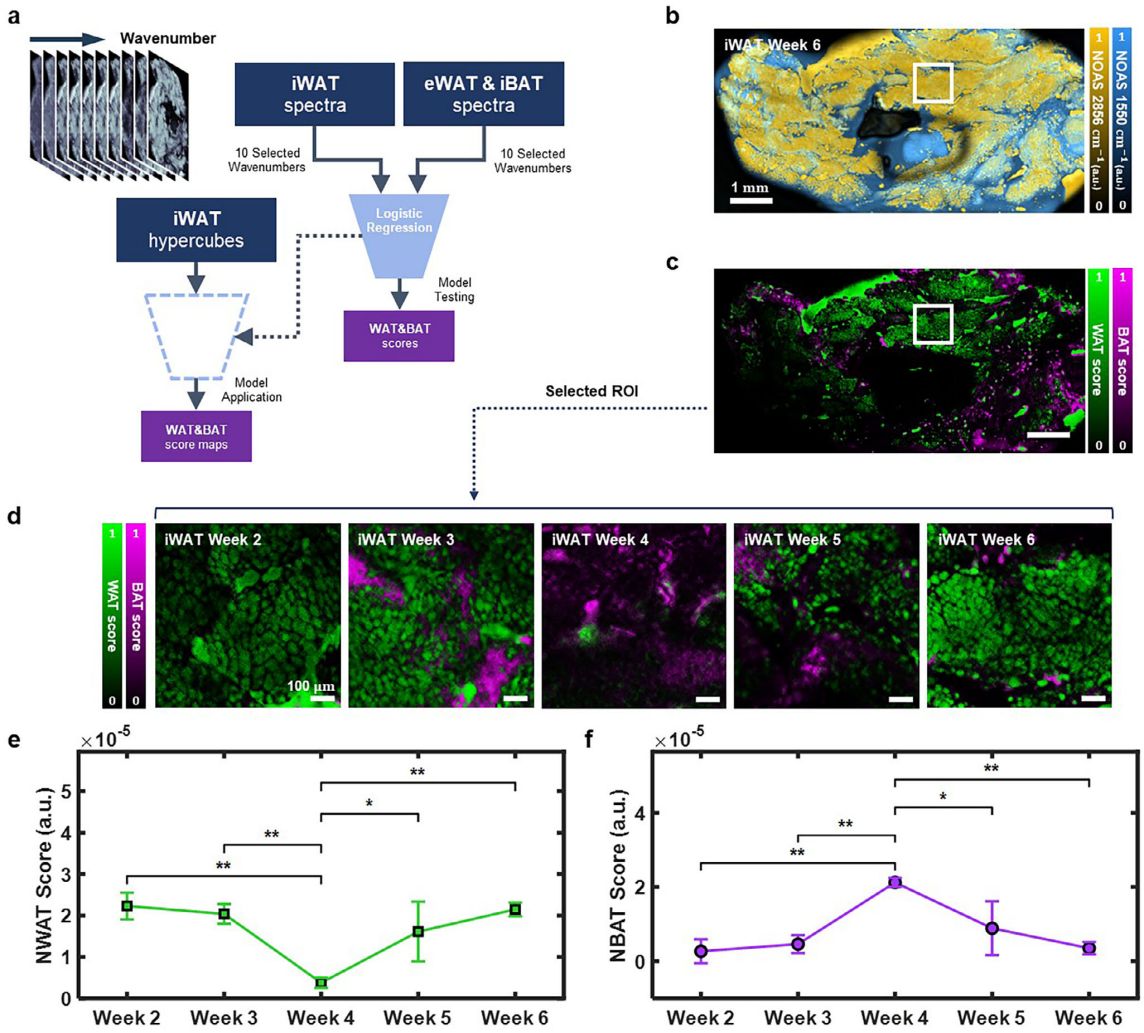
Quantitative spatial analysis of postnatal tissue remodeling in inguinal white adipose tissue. (a) Flowchart of the quantitative spatial analysis tool (Q‐SAT) used for pixel‐wise digital scoring the level of WAT or BAT similarity of iWAT. (b) Merged depth selective OA micrograph at 2856 cm^−1^ (yellow) and 1550 cm^−1^ (cyan) for a large FOV (5  ×  10 mm^2^) collected during iWAT postnatal week 6. (c) WAT and BAT score mapping results for b. (d) Digital scoring map for a 1  ×  1 mm^2^ FOV area during postnatal development (Week 2–6) in iWAT. (e,f) Mean normalized WAT and BAT (NWAT, NBAT) scores for the 1 × 1 mm^2^ FOVs images obtained from all measurements (Week 2: *n* = 2 mice; Week 3–6: *n* = 3 mice). The error bars indicate SD. Results in e,f show statistical analysis for comparisons across postnatal weeks, one‐way ANOVA with Bonferroni‐corrected post‐hoc tests, of NWAT and NBAT scores per mouse for each tissue type (see Methods: Statistical Analysis).

Figure 3a depicts the flowchart of Q‐SAT. The underlying method of Q‐SAT consists of a logistic regression model trained on the depth‐selective OA signal intensities of 1) adipocytes from eWAT and iBAT, and 2) further tissue structures located in the iWAT, including ECM, lymph nodes, connective tissue, water, etc. The model segments and scores adipocytes with respect to spectral features related to WAT or BAT based on 10 wavenumbers (2856, 1632, 1550, 1454, 1376, 1238, 1134, 1086, 1046, and 990 cm^−1^), selected after analyzing spectra obtained on each tissue structure, see Methods for details. The trained model was then applied to segment and digitally score adipocyte features in hyperspectral iWAT images. Similar to the previously discussed tissue scoring, the digital scoring is related to adipocyte features that are either corresponding to WAT or BAT. Figure 3b shows an overlay of the OA absorption map of iWAT based on two vibrational contrasts (yellow: 2856 cm^−1^, cyan: 1550 cm^−1^) at postnatal week 6 from a large FOV (5 × 10 mm^2^). Below the OA absorption map, a digital scoring map of the same FOV is visualized (Figure 3c). To monitor tissue remodeling in iWAT during postnatal development, we applied Q‐SAT to images from all postnatal stages of iWAT in a small FOV (1 × 1 mm^2^), as shown in Figure 3d, and plotted the digital contrast in a more intuitive way in Figure 3e,f. to quantitatively compare WAT and BAT scores produced by Q‐SAT during postnatal development (Week 2: *n* = 2 mice; Week 3–6: *n* = 3 mice). Thereby, as expected, we observed that the relative overall WAT score of iWAT decreased during the browning phase of postnatal weeks 2–4, while the relative overall BAT score increased. The inverted trend was observed during the whitening phase of the postnatal weeks 4–6. To quantitatively assess how NBAT score reflects thermogenic activation, we compared the mean NBAT score with the mean UCP1 fluorescence intensity from immunofluorescence in iWAT across the postnatal time points. For each postnatal week, we calculated the mean NBAT score across the Q‐SAT maps from three independent measurements and the corresponding mean UCP1 fluorescence intensity in the matched immunofluorescence images in Figure S3c. Pearson correlation analysis revealed a strong positive correlation between mean NBAT score and mean UCP1 fluorescence intensity (r = 0.95), indicating that the NBAT score closely tracks UCP1 protein level. (Figure S3d). Q‐SAT is used to score adipocytes along a WAT‐like and BAT‐like biochemical axis and to visualize their spatial distribution. In principle, the same approach could be extended to classify additional adipocyte subtype states with appropriate training labels and markers. Here we focus on quantifying the spatial organization of WAT‐like and BAT‐like phenotypes. To quantify spatial pattern beyond visual inspection, we computed a tile‐based heterogeneity metric by dividing each Q‐SAT score map into a 10 × 10 grid and calculating the coefficient of variation (CV) of tile‐mean NWAT and NBAT scores. A high CV indicates patchy regional enrichment, whereas a low CV indicates a more homogeneous spatial distribution. The resulting CV profiles indicate that postnatal remodeling proceeds as a spatially localized process, with WAT‐like and BAT‐like phenotypes forming a mosaic of enriched regions rather than a uniformly mixed intermediate state. (Figure S4). With Q‐SAT we unveiled the spatially heterogeneous appearance of adipose tissue remodeling while simultaneously avoiding the selection bias of localized spectral analysis associated with manual structure selection.

## 3 Discussion

In this study, we demonstrated that MiROM can quantitatively monitor the intracellular changes of biomolecules in fresh unprocessed fat tissues while preserving the native tissue architecture in a depth‐selective and label‐free manner. In particular, intracellular multi‐spectral imaging and spectral analysis using MiROM enabled comparison and differentiation of the morphological and molecular hallmarks of eWAT and iBAT, as well as the monitoring of dynamic WAT remodeling. We thus developed a new methodology that can aid in the identification and characterization of adipose tissue plasticity and heterogeneity, providing a powerful tool for studying tissue physiology and pathology in situ.

With MiROM, we observed distinctive morphological and molecular differences between adipocytes in eWAT and iBAT. For instance, as expected from H&E‐based histology, we observed that eWAT‐adipocytes are ca. 3.3 times larger than iBAT‐adipocytes and that the lipid contrast (at 2856 cm^−1^) from eWAT‐adipocytes is ca. 1.7 times higher than the lipid contrast originating from iBAT‐adipocytes [42]. Furthermore, as expected from western blot measurements, we observed that the protein contrast (at 1550 cm^−1^) in iBAT‐adipocytes is ca. 3.4 times higher than in eWAT‐adipocytes. This confirms that, compared to iBAT, eWAT is a lipid‐rich and protein‐poor tissue, mostly due to its larger size of lipid droplets and a lower number of mitochondria, as shown in previous studies on mitochondrial abundance and lipid droplet morphology [42]. Accordingly, the strong amide II absorption contrast at 1550 cm^−1^ provides a direct, label‐free readout of the higher intracellular protein fraction that accompanies mitochondrial enrichment in brown or beige adipocytes, whereas eWAT spectra are dominated by neutral lipid signatures. Additionally, by analyzing the intrinsic molecular and morphological hallmarks of eWAT and iBAT obtained with MiROM, we were able to monitor iWAT browning during postnatal weeks 2–4 in mice and its subsequent re‐whitening during postnatal weeks 4–6. This observation was confirmed by western blot, H&E staining, and whole‐mount immunofluorescence staining (Figure S4). Moreover, beyond qualitative imaging, we assessed the spatial distribution of adipocyte types in different postnatal weeks during the adipose tissue remodeling process and observed substantial heterogeneities with respect to different postnatal weeks based on pixel‐wise tissue scoring with Q‐SAT. Since Q‐SAT uses only a low number of spectral points (10 wavenumbers), it significantly increased the hyperspectral image acquisition speed (50‐fold acceleration). Collectively, MiROM offers technology for intracellular analysis of adipose tissue in a label‐free and non‐destructive manner by enabling the assessment of the native tissue architecture and intrinsic biomolecules while avoiding time‐consuming tissue preparation, including fixation and staining. Beyond describing postnatal remodeling, the tile‐based CV (spatial heterogeneity analysis) provides a scalable framework for future application of MiROM/Q‐SAT. First, it can be used as a quantitative endpoint to compare spatial remodeling patterns on physiological or pharmacological interventions. Second, tile‐level phenotypic maps can be used to nominate regions of interest for further analysis, such as correlating BAT‐like adipocyte enrichment with microenvironmental features (e.g., proximity to vasculature or nerves). In future work, we could extend this framework to resolve finer adipocyte subtype categories with additional biochemical markers and complementary measurements.

Here, focused on characterizing overall lipid and protein content in adipocytes during adipose tissue remodeling. However, since lipid composition and the number of mitochondria play a critical role in the metabolic function of adipocytes, for a better understanding of adipose tissue remodeling, combining MiROM readouts with mass spectroscopy‐aided in‐depth lipidomics and proteomics could reveal the sample's single‐biomolecular composition. Additionally, we anticipate that the combination of hyperspectral imaging using MiROM aided with Q‐SAT and mass spectrometry cross‐validation will provide a quantitative approach for assessing biomolecules of adipocytes while providing native morphological information.

Furthermore, while Q‐SAT significantly reduced the required number of spectral points for the assessment of the spatial distribution of adipocyte types, the hyperspectral imaging acquisition time is still limited by the mechanical properties of MiROM, i.e., the speed and acceleration of the motorized stages used for point‐by‐point raster scanning. As the number of imaged wavenumbers increases, the longer the data acquisition time, and thus a tradeoff arises between tissue scoring quality and time required for imaging. Hence, the number of wavenumbers used for tissue scoring can be parametrically chosen in accordance with the desired imaging speed and output quality. To investigate the influence of reducing the imaged wavenumbers on the output quality, we compared the tissue scoring method based on 10 wavenumbers with a 2‐wavenumber setting by training and comparing models using tissue scans, as shown in Figure S5. The investigation revealed that both approaches enable visualization of tissue browning and whitening over time. Furthermore, the normalized tissue scores quantitatively exhibit transient tissue features during postnatal development for both methods, as shown in Figure S5b,c,e,f. A direct comparison of the two approaches qualitatively reveals subtle intensity differences in the scoring maps. Furthermore, the direct comparison shows that the 2‐wavenumber approach is susceptible to artifacts related to misclassified tissue components, particularly connective tissue, which is incorrectly scored as brown adipose tissue. Hence, the 10‐wavenumber method was chosen for display in Figure 3, since although the 2‐wavenumber method achieves tracking the tissue remodeling, the 10‐wavenumber method achieves improved scoring fidelity. To address the limitations associated with long imaging times, new strategies to achieve fast image acquisition speed are currently being implemented in our lab. These involve galvo‐ or MEMS (micro‐electro‐mechanical systems)‐mirrors, simultaneous acquisition of OA transients with multiple wavenumbers, increasing laser repetition rate up to 2MHz, and sparse data acquisition combined with Bayesian image reconstruction [43].

In summary, we characterized molecular hallmarks of eWAT and iBAT and monitored intracellular molecular and morphological changes of adipocytes during postnatal transient adipose tissue remodeling using MiROM. As a result, we identified novel biochemically distinguishable characteristics between WAT and BAT, developed a new methodology that can aid in the identification and monitoring of the adipose tissue browning and whitening process and gained insights into the metabolic plasticity of adipose tissue at the intracellular level. With Q‐SAT, we further obtained spatial assessments of beige adipocyte development at the tissue level. Beyond monitoring postnatal adipose tissue remodeling via leveraging previously unrecognized biochemical features that fundamentally differentiate tissue identity, as presented here, MiROM equipped with Q‐SAT could support evaluation of metabolic disease interventions by providing means for objective, spatially resolved metabolic imaging in tissues. Specifically, MiROM/Q‐SAT could quantify when, where and to what extent BAT‐like adipocytes emerge within WAT in response to pharmacological or environmental browning stimuli, enabling standardized assessment in preclinical obesity and diabetes models [44]. In addition, MiROM/Q‐SAT could be extended to other lipid‐driven pathologies such as alcoholic and non‐alcoholic fatty liver disease. Here, it could complement conventional histology by providing a label‐free quantitative, spatially resolved measure of hepatic lipid accumulation (steatosis) across a field of view, which may reduce reliance on stain‐dependent workflows and observer‐dependent grading while preserving spatial information that is lost in bulk lipid assays [45].

## 4 Methods

### 4.1 Animal

Mice housing and euthanasia were conducted in accordance with the German Animal Welfare Act. Animals were maintained under standard conditions in individually ventilated cages at the mouse facilities of the Technical University of Munich, with food and water provided ad libitum. Animals were euthanized exclusively for the purpose of organ and tissue collection for scientific analyses, without any experimental intervention or procedure that could cause pain, suffering, or distress beyond the act of killing itself. In accordance with § 7 of the German Animal Welfare Act (TierSchG) in conjunction with §§ 4, 7a, and 8 of the TierSchG, these procedures do not constitute an animal experiment and therefore do not require approval by an ethics committee or the assignment of an approval or accreditation number.

### 4.2 MiROM Configuration and Measurement Process of Adipose Tissue

MiROM in transmission mode is shown in Figure 1a. A pulsed QCL (MIRcat, Daylight Solutions) was used as a mid‐IR excitation source for OA signal generation. Since the QCL spectral range covered 3.4–11 µm (2932–909 cm^−1^) and the full width at half maximum (FWHM) of the spectral linewidth was <1 cm^−1^, biomolecular specificity was able to be achieved during the measurement. The focused mid‐IR laser beam (Repetition rate: 100 kHz, pulse duration: 20 ns), focused by 0.5 NA reflective objective (36 ×, Newport Corporation), excited the sample located on top of the mid‐IR transparent ZnS window (Crystal) of the custom‐designed metallic petri dish. The tissue sample was placed on the ZnS window after removing the superficial buffer solution from the surface of the tissue to decrease the mid‐IR attenuation, which is driven by the strong mid‐IR absorption of water. An acoustically transparent plastic membrane covers the tissue with ultrasound gel to isolate the tissue from the coupling media (Deionized water) while it is feasible to transfer the generated acoustic wave from the focal spot of the mid‐IR beam. The generated OA signal has been acquired by a focused ultrasound detector (Imasonic and Sonaxis), which has a 20 or 25 MHz central frequency, immersed in coupling media. The raw OA signals were amplified with a 63 dB low‐noise amplifier (MITEQ) and filtered with a 50 MHz low‐pass filter (Mini‐circuits). Filtered OA signals recorded by 200 MS/s on a data acquisition (DAQ) card (Gage Applied). To avoid the interference of CO_2_ and water vapor, the optical beam path has been covered by the purged inert gas chamber with dry N_2_ gas.

The lateral and axial resolution of MiROM has been experimentally assessed by measuring the point‐spread‐function (PSF) of a polystyrene sphere with a size of 1 µm at 2850 cm^−1^ (Lateral resolution: 5.3 µm, axial resolution: 42.2 µm). The detailed optical performance of MiROM is reported elsewhere [29].

### 4.3 Depth‐Selective Analysis and Lateral Segmentation of Adipocytes

To compare the interior characteristics of adipocytes within the same depth range, we utilized a time dependency of the OA signal. Since the acoustic wave allows us to estimate the point of signal generation from signal arrival time, we could deduce axial information of adipocytes. To analyze the OA signal at the specific depth range, we acquired the absolute (Abs.) of the OA analytic signal from the OA transient (A‐line). All OA analytic signals were obtained from the OA transients, which were interpolated using cubic interpolation. We defined the minimum size of the time window (depth range) as 7.5 µm (1 sampling time point; sampling rate: 200 MS/s, estimated acoustic wave speed at soft tissue: 1500 m/s). For the intracellular comparative analysis of each tissue, we acquired each depth‐selective intensity of the mid‐IR absorption map (micrograph) and spectra based on the area under the curve of the Abs. of the OA analytic signal from the defined time window. Since the primary content of adipocytes is lipid droplets, we defined an intracellular time window based on maximum amplitude depth in 2856 cm^−1^.

Additionally, to localize adipocytes from extracellular components, we also laterally segmented the mid‐IR absorption map through intensity thresholding. We obtained segmentation masks from each micrograph with the pixels with intensities higher than the first quantile (25% of data) of iBAT's OA contrast at 2856 cm^−1^.

### 4.4 MiROM Image Acquisition and Processing

Since the system was designed based on the co‐alignment of the mid‐IR excitation beam and focused ultrasound detector, to obtain the mid‐IR absorption map, the OA transients have been acquired by point‐by‐point raster scanning pattern of *x*‐*y* motorized stage (Physik Instrumente). The optical and acoustical focal planes have been adjusted by the *z*‐axis mechanical stages, and the ultrasound detector and reflective objective are individually mounted. Each pixel intensity of the micrographs has been obtained by the chosen wavenumbers from 2932–2770 cm^−1^ and 1738–909 cm^−1^ at the corresponding position assigned by the *x*‐*y* motorized stage. We acquired intensity in two ways from the OA transient: peak‐to‐peak intensity of OA transient (MAP) or depth‐selective intensity, which is mentioned in the previous section. At each pixel position, 50 OA transients have been acquired (since the pulse repetition rate is 100 kHz, the pixel acquisition time is 500 µs.). The averaged intensity of 50 OA transients has been formed as the intensity of each pixel. Micrographs are obtained at a FOV of 500 × 500 µm2, 1 × 1 mm^2^ with 2 or 5 µm step sizes (500 × 500 µm^2^ (step size – 2 µm): 13 min, 1 × 1 mm^2^ (step size – 2 µm): 40 min, 1 × 1 mm^2^ (step size – 5 µm): 8 min per single wavenumber).

For the contrast enhancement of the micrograph, to analyze the structural details, two image post‐processing steps have been utilized on all MiROM micrographs in the figures displayed in the manuscript: normalized square intensity of the OA signal (OAS), $\mathrm{NOA}{S_{x}}^{2}={\left(\frac{\mathrm{OA}S_{x}-\min\left(\mathrm{OA}S_{\mathrm{FOV}}\right)}{\max\left(\mathrm{OA}S_{\mathrm{FOV}}\right)-\min\left(\mathrm{OA}S_{\mathrm{FOV}}\right)}\right)}^{2}$, and contrast limited adaptive histogram equalization (CLAHE). Processed micrographs acquired from 2856 cm^−1^ have been utilized to analyze the size of adipocytes from each adipose tissue, respectively. The adipocyte boundaries were defined based on the contrast difference between adipocytes and the extracellular structure of adipocytes at 2856 cm^−1^, and the size of adipocytes was calculated based on the Cell Profiler 4.0 software.

### 4.5 MiROM Spectra Acquisition and Processing

The localized mid‐IR absorption spectra were acquired while tuning the pulse tunable QCL with a step size of 2 cm^−1^ from 2932–2770 cm^−1^ and 1738–909 cm^−1^ at a single focal spot (∼5.3 µm) positioned at corresponding localized locations from the sample (e.g., near the center of each identified adipocyte), so that each spectrum represents a localized single‐cell measurement. To enhance the accuracy of the spectral analysis of adipocytes, 10 000 OA transients per wavenumber at each adipocyte have been obtained to increase the signal‐to‐noise ratio (SNR). The spectral acquisition time of each adipocyte is ∼8 min. The raw OA intensity profile across wavenumbers obtained at MiROM spectral analysis, displayed in the manuscript, was depth‐selective intensity.

Since the OA signal has been affected by the daily response of the system, such as laser fluctuation, alignment of optics, and acoustic detector, to compensate for the slight variations on the laser emission profile of the QCL, we obtained OA spectral intensity across wavenumbers by correcting the sample's raw OA intensity profile (Intensity: depth‐selective intensity) dividing by OA intensity profile (Intensity: peak‐to‐peak value of OA transient) of the reference sample (carbon adhesive tape). Since the carbon adhesive tape shows broadband absorption at the emission range of the QCL, it allows the measurement of the laser emission profile of the QCL.

### 4.6 Machine‐Learning‐Based Digital Staining and Tissue Scoring

Combining and generating a metric to describe the spectral hallmarks of BAT and WAT enables quantitative tracking of the tissue remodeling presented in this work. To exhibit the temporal development of browning and whitening of iWAT, we, therefore, implemented a machine‐learning approach to quantify tissue properties based on spectral information. The workflow used for modeling was to train a logistic regression model based on the reference spectra extracted from eWAT and iBAT while labeling the tissue types (WAT&BAT) of the corresponding spectra. Applying the trained model to the spectra collected from adipocytes of individual postnatal stages and extracting the predicted probabilities for WAT&BAT allows us to statistically assess the predicted probabilities used to score spectral properties over the postnatal stages. In a subsequent approach, we selected 10 wavenumbers as model features determined using PCA containing information about the spectra's corresponding tissue types and trained another logistic regression model based on the selected wavenumbers to ultimately apply the model to hyperspectral tissue images for digital staining. Therefore, we additionally included spectra from iWAT corresponding to additionally labeled structures apparent in the images, such as ECM, lymph nodes, water, and void area, to enable discrimination of the model between spectral hallmarks related to adipocytes and other structures. Applying the trained model to the hyperspectral images acquired based on the 10 excitation wavenumbers and extracting the pixel‐wise prediction probabilities enables digital staining of spectral hallmarks related to BAT and WAT in a spatial context. Similar to the statistical evaluation of the spectral scoring, the scores from the images taken from iWAT were statistically assessed over all pixels to quantify the overall tissue score and thus track the tissue remodeling process over time. The overall metric for the normalized tissue scores *S*_WAT_ and *S*_BAT_ extracted for each hyperspectral image in every postnatal week was computed based on the pixel‐wise model outputs WAT(*x*, *y*) and WAT(*x*, *y*) integrated over the entire image, i.e.,

$$S_{WAT}=\frac{\sum_{x,y}WAT\left(x,y\right)}{\sum_{x,y}\left[WAT\left(x,y\right)+BAT\left(x,y\right)\right]}$$

and,

$$S_{WAT}=\frac{\sum_{x,y}BAT\left(x,y\right)}{\sum_{x,y}\left[WAT\left(x,y\right)+BAT\left(x,y\right)\right]}$$

to put the WAT&BAT scores concerning the overall content related to structures with spectral hallmarks of adipocytes. The normalized tissue scores *S*_WAT_ and *S*_BAT_ enable digital staining of spectral properties related to WAT&BAT and thus constitute a statistical metric to track the tissue remodeling process.

To further characterize the working principle of Q‐SAT, we analyzed the contributions of the wavelengths for the scoring targets. Based on the Shapley Additive Explanations (SHAP) (Figure S6a–c) and their mean absolute values (Figure S6d–f), we found that the wavenumbers at 990 and 1238 cm^−1^ are most instrumental in discriminating between white adipose tissue (WAT), brown adipose tissue (BAT), and connective tissue. Based on this finding, we investigated the use of only two wavenumbers for tissue scoring, as acquiring fewer wavenumbers reduces imaging time. Therefore, we compared the 10‐wavenumber‐based tissue scoring results with those obtained using a 2‐wavenumber‐based approach with the wavenumber combinations (990, 1238 cm^−1^) and (1550, 2856 cm^−1^). Figure S6g–i shows the Receiver operating characteristic (ROC) curves evaluated on the test dataset, which indicate high fidelity in all three approaches, as the area under the curve (AUC) is above 0.95 in every case, although highest in the 10‐wavenumber‐based method (approximately 0.99). However, comparing the three models with respect to the full‐image tissue scoring results based on a controlled WAT sample, potential artifacts become visible. Figure S6j–l shows that for the 2‐wavenumber approach using (1550, 2856 cm^−1^) connective tissue erroneously expresses high BAT scores, while (990, 1238 cm^−1^) yields overly low scores for white adipocytes in comparison with the 10‐wavenumber approach.

### 4.7 Feature Extraction and Data Preprocessing

Tissue scoring requires features to be extracted from OA signals and preprocessed for training and application of the machine learning model. The raw signals acquired using MiROM are OA transients carrying structural information about the absorption coefficients in the sample. The digital staining model uses depth‐selective intensities as features derived from the A‐lines according to the ‘Depth‐selective analysis and lateral segmentation of adipocytes’ section. The extracted intensity values are then divided by the corresponding carbon spectrum intensities to compensate for the laser emission profile. For training the spectral scoring, the spectra were normalized to the uniform spectral norm by dividing each spectrum $s\;={\left[\begin{array}{ccc} I(\lambda_{0}) & \text{…} & I(\lambda_{N}) \end{array}\right]}^{T}$ composed by OA spectral intensity values for each wavenumber λ by the spectral L2‐norm, i.e.

$$\;s_{\mathrm{normal}}=\frac{s}{{\left(\sum_{i}I{\left(\lambda_{i}\right)}^{2}\right)}^{-1}}\;,$$

to remove intensity drifts in the system and only account for the spectral shape to determine the scoring. While *N*  =  502 for the spectra taken at adipocytes, the normalization was applied analogously to the 10‐wavenumber hypercubes for each datapoint (pixels in the hyperspectral images) individually, where *N*  =  10. In order to ensure convergence and high accuracy of the algorithm to minimize the objective function given by the categorical cross‐entropy of the training dataset, the features were scaled using standardization, i.e.

$$I_{\mathrm{scaled}}\;\left(\lambda \right)=\frac{I\left(\lambda \right)-\mu_{\lambda }}{\sigma_{\lambda }}\;,$$

where μ_λ_ are the mean and σ_λ_ the standard deviation (SD) of all intensities for the wavenumber λ contained in the training dataset. Scaling unseen data, which are not part of the training dataset, was done analogously using μ_λ_ and σ_λ_ derived from the training data.

### 4.8 Adipoclear

Whole Tissue Clearing was performed following the Adipo‐Clear method published by Chi et al. in 2018 [46]. In short, whole iWAT pads were fixed with 4% paraformaldehyde in PBS for 24 h at room temperature. Fat pads were dehydrated using a series of methanol gradients in glycine buffer. Fat pads were delipidated in 100% dichloromethane, washed with 100% methanol, and subsequently bleached in a 5% H_2_O_2_ solution in methanol. After rehydration in a reverse methanol/glycine buffer gradient, fat pads were stained against UCP1 (Abcam, ab23841) at a 1:200 dilution containing Heparin and Triton X‐100 for one week. Fat pads were then washed and incubated for another week with a multi‐rAb CoraLite Plus 647‐Goat Anti‐Rabbit 647 secondary antibody (Proteintech, RGAR005) at a 1:500 dilution. Lint was gently removed from the fat pads under a dissection microscope. Fat pads were then embedded in a 1% Agarose in PBS solution to stabilize their morphology. Embedded fat pads were again dehydrated in a methanol gradient and delipidized in 100% DCM. Samples were incubated overnight in ethyl cinnamate for refractive index matching and analyzed by fluorescent microscopy (Zeiss, LSM 700).

### 4.9 Histology

iWAT was isolated and immediately fixed in 4% paraformaldehyde for 24 h at room temperature. Tissues were then dehydrated using an ethanol gradient and embedded in paraffin. Tissues were sectioned into 5 µm slices and stained with hematoxylin and eosin. Slices were analyzed using an EVOS XL Core Imaging System.

### 4.10 Tissue Preparation for MiROM Imaging

Whole adipose tissue pads were carefully isolated from FVB/NJ mice. All animal experiments were conducted in accordance with local legislation and in compliance with the ARRIVE guidelines. eWAT and iBAT pads were isolated from 6‐week‐old mice, while iWAT pads were isolated from mice aged between 2 and 6 weeks. Tissues were lightly fixed for 4 h in IC fixation buffer (eBioscience, 00‐8222‐49) at room temperature. After fixation, tissues were stored in PBS at 4°C until the day of measurement.

### 4.11 Western Blotting

Whole iWAT pads were isolated and snap‐frozen in liquid nitrogen. For protein isolation, 100–200 µL RIPA buffer was added to each whole fat pad. Fat pads were homogenized for 20 s using an UltraTurax T10 (IKA). The resulting homogenate was centrifuged at 4°C for 10 min at 14 000 g. The supernatant was collected, and protein concentration was measured using the Pierce BCA Protein Assay (Thermo Scientific, 23225). 30ug of Protein was loaded and then separated on a 12.5% PAGE Gel. Proteins were blotted onto a nitrocellulose membrane for 1h at 100 mA using a SemiDry blotting approach (Analytik Jena, Biometra Fastblot). The membrane was blocked with 5% BSA for 90 min at room temperature and then incubated in a 1:2000 rabbit anti‐UCP1 (Abcam, ab23841) and 1:5000 mouse anti‐Beta‐Actin (Proteintech, 66009‐1‐Ig) primary antibody solution overnight at 4°C. After multiple washes, secondary antibodies against rabbit and mouse (LICOR, 926—32 211 and 926—68 070) were added at 1:20 000 dilutions for 90 min at room temperature. Protein signals were detected using the 700 and 800 nm channels of a LICOR Odyssey FC Imager.

### 4.12 Statistical Analysis

Statistical analysis and figure preparation were performed using MATLAB 2021a. Adipocyte regions were segmented using the data‐processing workflow described in the Depth‐selective analysis and lateral segmentation of adipocytes, and adipocyte size and OA contrast were extracted within the resulting adipocyte masks. For Q‐SAT analysis, pixel‐wise NWAT and NBAT scores were computed from the hypercube with selected wavenumbers described in the Machine‐learning‐based digital staining and tissue scoring. All quantitative data are reported as mean±SD, and outliers were not removed. Statistical significance was defined as α = 0.05 (*p* < 0.05 considered significant), with asterisks indicating the level of significance (^*^ *p* < 0.05, ^**^ *p* < 0.01, ^***^ *p* < 0.001, ^****^ *p* < 0.0001). For two‐group comparison (e.g., eWAT and iBAT), inferential statistics were performed per mouse using an unpaired two‐sample *t*‐test. For multi‐group comparisons during postnatal remodeling (iWAT Week 2–6), inferential statistics were performed per mouse using one‐way ANOVA with postnatal week as a between‐subjects factor, followed by Bonferroni‐corrected post‐hoc comparisons. Results in Figure 1f,g represents the mean value per mouse (eWAT and iBAT: *n* = 7 mice per tissue type). Results in Figures 2e,f and S2d are based on the mean value per mouse (eWAT and iBAT: *n* = 7 mice per tissue type; iWAT Week 2–6: *n* = 5 mice per postnatal week). Results in Figure 3e,f are derived from the mean value per mouse (Week 2: *n* = 2 mice; Week 3–6: *n* = 3 mice). To visualize biological variability, cell‐ and pixel‐level data (overall adipocyte size, OA contrast, and NWAT&NBAT scores) are presented as box plots in Figure S7.

## Author Contributions

M.K., A.W., Y.L., and M.A.P. conceived and designed the study. M.K., A.W., and A.P. performed the experiments. M.K. and C.B. performed spectral data analysis. M.K., A.W., and C.B. prepared the figures. C.B. developed a digital scoring method. A.W. performed validation measurements of western blot, H&E staining, and whole‐mount immunofluorescence staining. V.N. provided support on optoacoustic detection. M.K. provided critical resources related to mouse maintenance and tissue preparation. M.K., A.W., C.B., Y.L., and M.A.P. wrote the manuscript. Y.L. supervised tissue acquisition and remodeling experiments. M.A.P. supervised the acquisition of OA images and spectra on mice tissues. Y.L. and M.A.P. supervised the whole study. All authors read and edited the manuscript.

## Conflicts of Interest

V.N. and M.A.P. are founders and equity owners of sThesis. V.N. is a founder and equity owner of Maurus OY, iThera Medical GmbH, Spear UG, and I3 Inc. The other authors declare no competing interests. A patent application (WO 2019 149 744 A1) licensed to sThesis GmbH, relevant to the technology discussed in this paper, has been filed.

## Supporting information




**Supporting File**: advs74592‐sup‐0001‐SuppMat.docx.

## Data Availability

The data that support the findings of this study are available from the corresponding author upon reasonable request.
