# Supplementary material for: Single‐Cell Metabolic Imaging and Digital Scoring of Fat Tissue Remodeling by Label‐Free Metabolic Microscopy
Source: Adv Sci (Weinh). 2026 Mar 2;13(28):e13042. doi: 10.1002/advs.202513042 (PMC13185827; doi:10.1002/advs.202513042)
Supplement: Supplementary file 1 — Supporting File: advs74592‐sup‐0001‐SuppMat.docx. [file ADVS-13-e13042-s001.docx]

**Supplementary Figures**


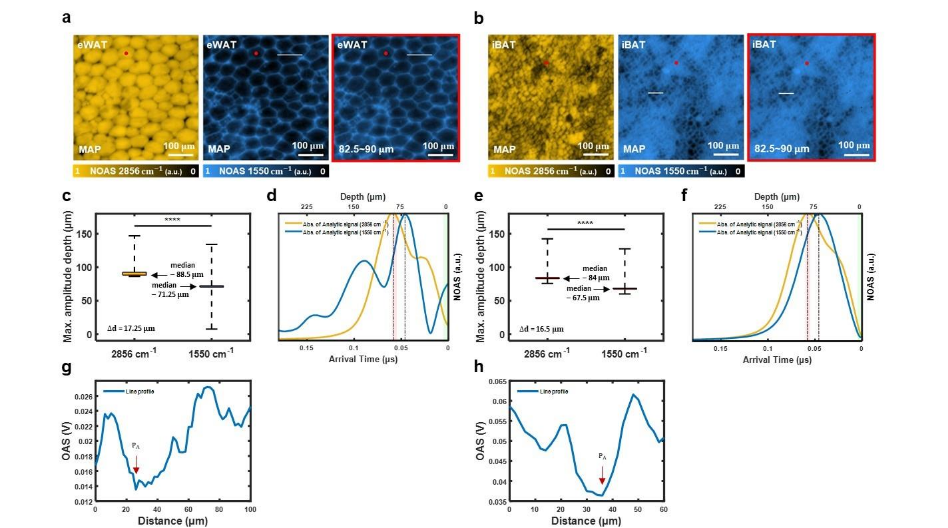


**Supplementary Figure S1. Comparison between maximum amplitude projection (MAP) analysis and the depth-selective analysis on eWAT by MiROM. (a, b)** MAP and depth-selective OA micrograph of eWAT and iBAT at 2856 and 1550 cm^-1^ channel. Depth-selective OA micrograph pixel intensity represents the area under the curve of the Absolute (**Abs.**) of the OA analytic signal within the selected depths. **(c, e)** Box plot showing the MAD at 2856 cm^-1^ and 1550 cm^-1^. Asterisks (****) denote the level of statistical significance (P<0.0001) analyzed by a two-sample t-test (**c**: eWAT, **e**: iBAT). **(d, f)** Abs. of OA analytic signal from 2856 and 1550 cm^-1^ (**d**: eWAT, **f**: iBAT). The red box indicates the time window (82.5~90 µm), which contains the depth of interest (MAD at 2856 cm^-1^; width: 7.5 µm). **(g, h)** Contrast line profiles, white line from at MAP OA micrographs in 1550 cm^-1^ (**g**: eWAT MAP OA micrograph, **h**: iBAT MAP OA micrograph).


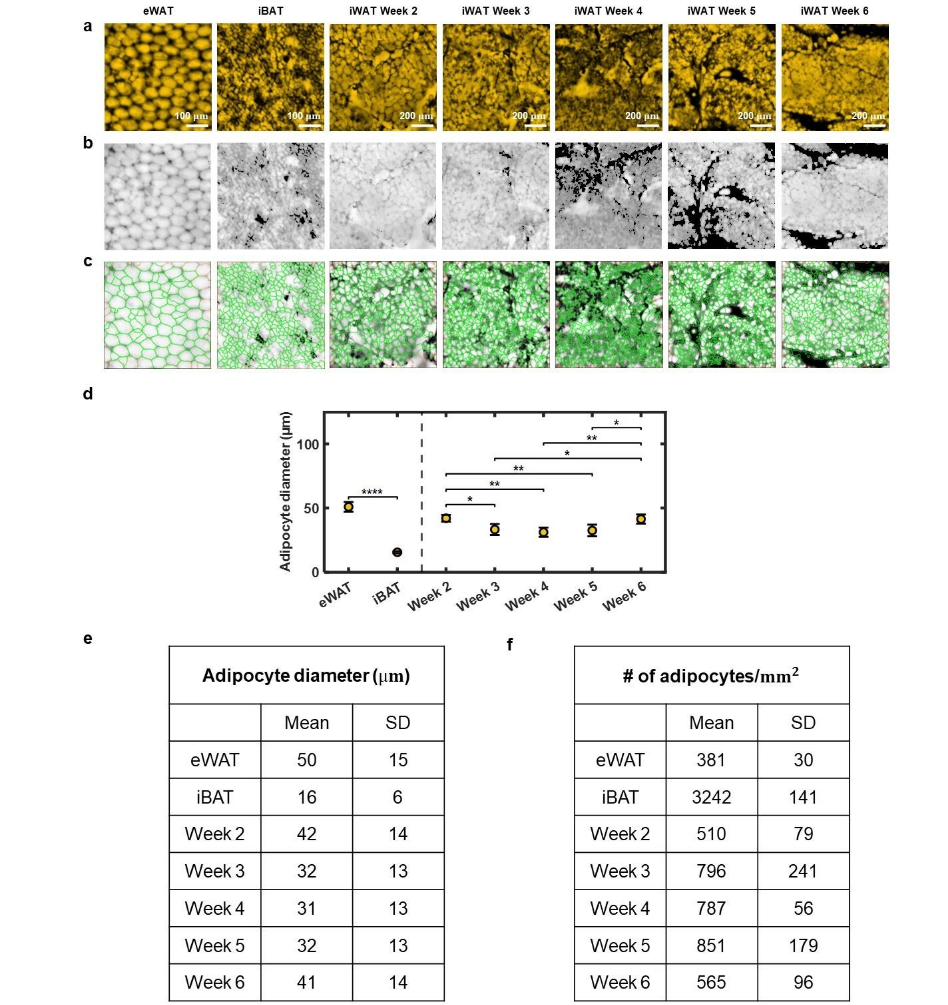


**Supplementary Figure S2. Intensity threshold-based masking results and adipocyte diameter and adipocyte density (adipocyte count per unit area) calculation at depth-selective OA micrograph. (a)** Depth-selective OA micrograph of eWAT, iBAT, and iWAT of postnatal weeks 2-6 at 2856 cm^-1^ channel. **(b)** Intensity threshold-based masking results of depth-selective OA micrographs of eWAT, iBAT, and iWAT of postnatal weeks 2-6 at 2856 cm^-1^ channel. Lateral segmentation based on intensity thresholding highlights the location of adipocytes. **(c)** Adipocyte segmentation produced by Cell Profiler 4.0 at selected eWAT and iBAT regions of interest (**ROI**). **(d)** Mean adipocyte size from ROI of eWAT, iBAT, and iWAT of postnatal weeks 2-6 (eWAT and iBAT: N = 7 mice for each tissue type; iWAT at postnatal weeks 2-6: N = 5 mice for each tissue type) with error bars indicating SD. **(e, f)** Mean and SD of adipocyte diameter and adipocyte count per unit area (mm^2^) of eWAT, iBAT, and iWAT of postnatal weeks 2-6. In **d**, for two group tissue comparisons (eWAT and iBAT), unpaired two-sample t-tests were used; for comparisons across postnatal weeks, using one-way ANOVA with Bonferroni-corrected post-hoc tests was used (see **Methods**: Statistical Analysis).


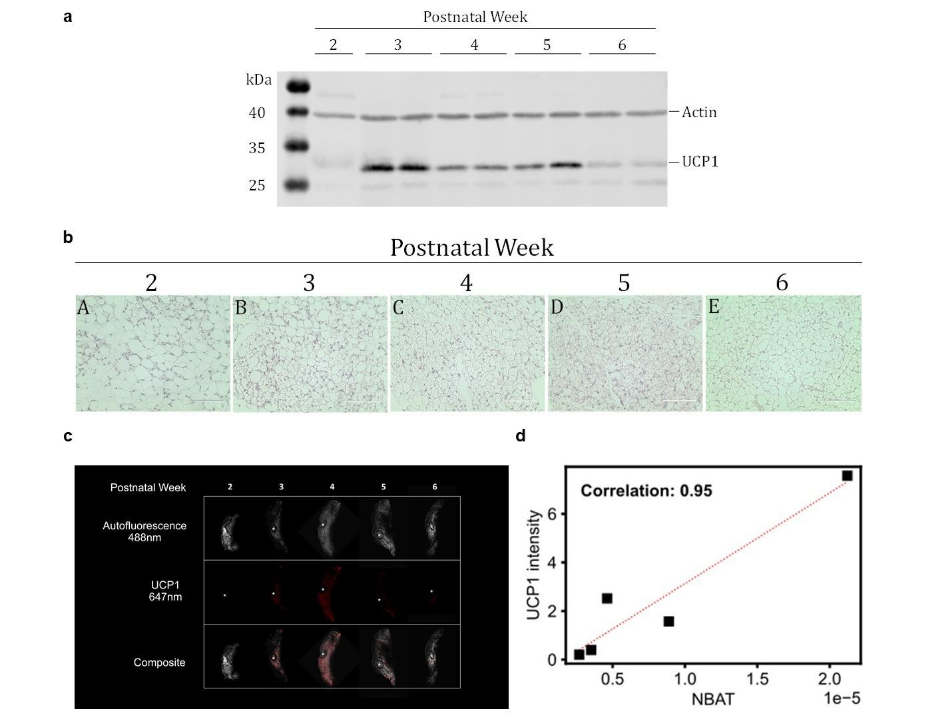


**Supplementary Figure S3. Biological validation of uncoupling protein 1 (UCP1) levels and morphological changes of adipocytes are transiently elevated in iWAT during postnatal development. (a)** Protein was isolated from iWAT during postnatal weeks 2-6. Immunoblots were stained against UCP1 (Abcam, ab23841) and beta-Actin (Proteintech, 66009-1-Ig). **(b)** Histological sections of iWAT from postnatal weeks 2-6 stained with hematoxylin and eosin (**H&E**) and depicted at 40x magnification. All sections were captured near the LN present in iWAT to ensure comparability between histological sections and MiROM measurements. **(c)** The panel depicts iWAT from postnatal weeks 2-6. Each image is a stitched optical section captured with a 5x objective. Each column depicts one iWAT sample with its autofluorescence in grey, an immunolabel against UCP1 in red, and their composite. The LN of each tissue is marked by *. **(d)** Correlation between mean NBAT score and mean UCP1 fluorescence intensity. Mean NBAT score obtained from all independent measurements (Week 2: N = 2 mice; Week 3-6: N = 3 mice) and mean UCP fluorescence intensity obtained from the matched immunofluorescence images in **c**.


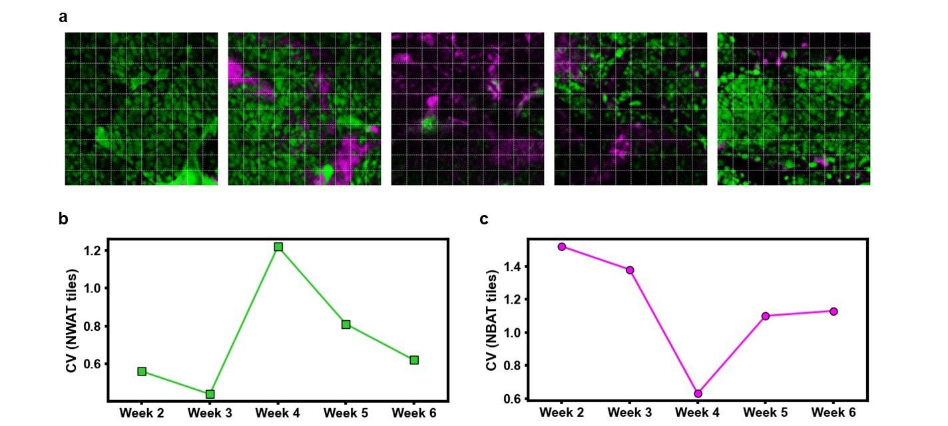


**Supplementary Figure S4. Tile-based spatial heterogeneity of Q-SAT NWAT and NBAT score maps in iWAT of postnatal week 2-6. (a)** Representative Q-SAT score overlays (NWAT score in lime green, NBAT score in magenta) reconstructed as 200

×*×*

200 maps and divided into a 10

×*×*

10 grid (dashed white lines; 20

×*×*

20 pixels per tile) to quantify regional variation in phenotype scores. **(b, c)** Coefficient of variation (CV) of tile-mean scores computed separately for NWAT (left, green squares) and NBAT (right, magenta circles) across postnatal weeks (computed from micrographs in **a**. Higher CV indicates greater patchiness (regional enrichment), whereas lower CV indicates a more spatially homogeneous distribution of the corresponding phenotype score.


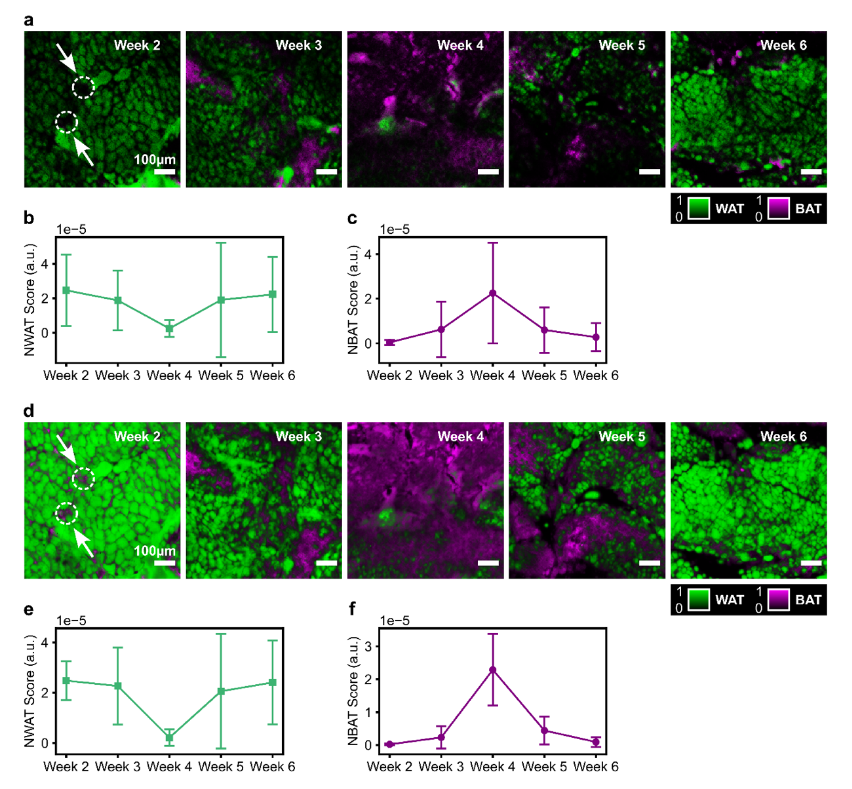


**Supplementary Figure S5. Comparing 10-wavenumber-based quantitative spatial analysis of adipose tissue with a 2-wavenumber-based approach. (a)** 10-wavenumber-based digital scoring map of inguinal white adipose tissue (iWAT) samples during postnatal development. The scores reflect spectral features associated with white adipose tissue (WAT) and brown adipose tissue (BAT). The marked areas highlight connective tissue for comparison with **d**. **(b-c),** Mean normalized WAT and BAT (NWAT, NBAT) scores for the tissue images shown in **a**. The error bars represent the standard deviation **c,** Explain. **d,** 2-wavenumber-based digital scoring map of iWAT samples during postnatal development. **(e-f)** Mean normalized WAT and BAT (NWAT, NBAT) scores for the tissue images shown in **d**.


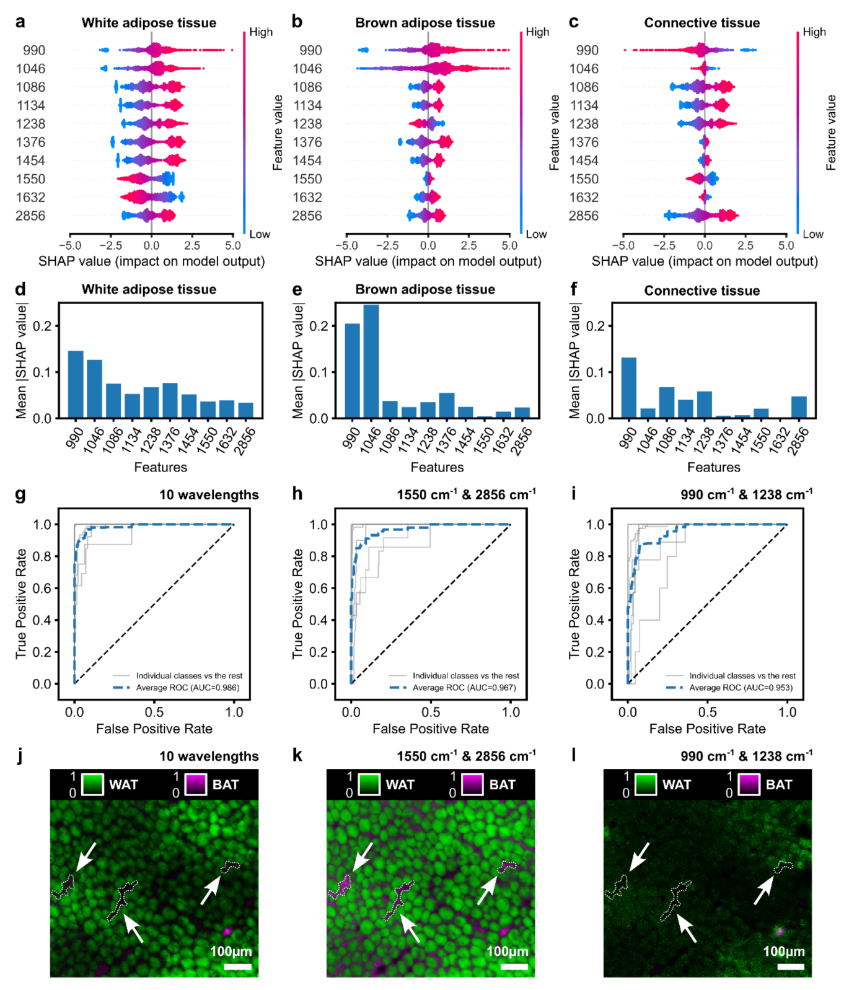


**Supplementary Figure S6. Quantitative spatial analysis model interpretation. (a-c)** Shapley Additive Explanations (SHAP) for pixel-wise segmentation of white adipose tissue (WAT), brown adipose tissue (BAT), and connective tissue. **(d-f)** Feature importance given by the mean absolute SHAP value for pixel-wise segmentation of WAT, BAT, and connective tissue. **(g)** Receiver operating characteristic (ROC) for tissue analysis based on the full wavelength set. The area under the ROC curve is given in the legend. **(h)** ROC for tissue analysis based on two-wavelength (1550 cm^-1^ and 2856 cm^-1^). **i,** ROC for tissue analysis based on two-wavelength (990 cm^-1^ and 1238 cm^-1^). **(j-l),** Digitally stained tissues using the full wavelength set (**j**) and the two-wavelength-based approaches, i.e., using 1550 cm^-1^ and 2856 cm^-1^ (**k**) and 990 cm^-1^ and 1238 cm^-1^ (**l**). Exemplary segmentation of connective tissue is visualized in all images for comparison.


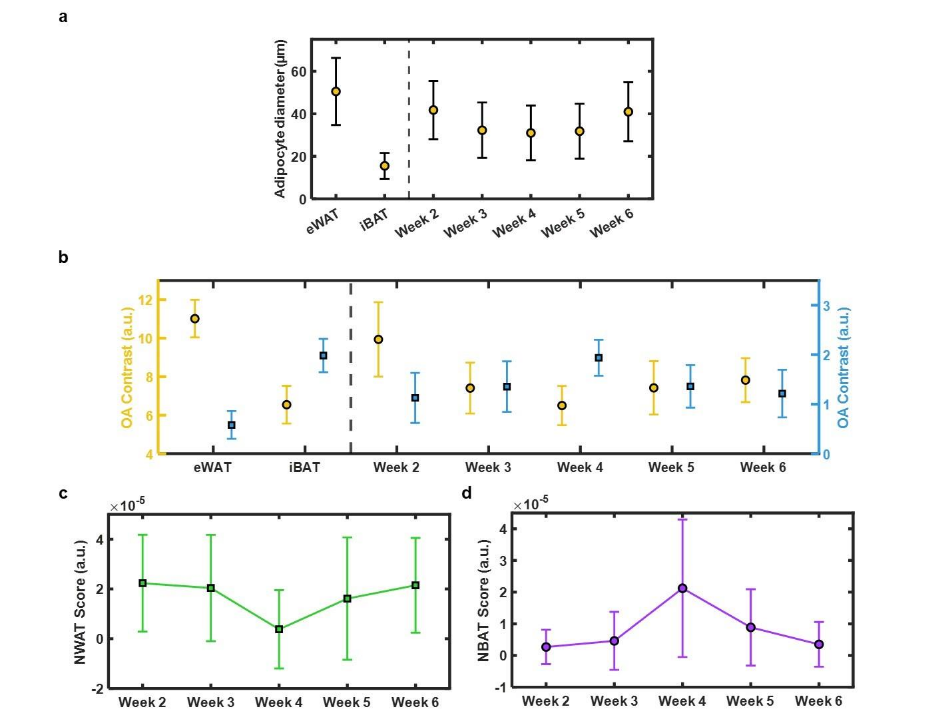


**Supplementary Figure S7. Descriptive summaries of overall adipocyte size, MiROM OA contrast, and Q-SAT NWAT and NBAT scores of eWAT, iBAT, and iWAT of postnatal week 2-6. (a)** Mean adipocyte diameter in eWAT, iBAT, and iWAT at postnatal week 2-6. **(b, c)** Mean OA contrast at 2856 and 1550 cm^-1^ of digitally isolated adipocyte area in eWAT, iBAT, and iWAT of postnatal week 2-6. **(c, d)** Mean NWAT and NBAT scores in iWAT of postnatal week 2-6, obtained from Q-SAT. Results are based on overall adipocyte size, OA contrast, NWAT&NBAT scores from every cell or pixel per tissue types. In **a, b**, each data point represents the mean value per tissue type (eWAT and iBAT: N = 7 mice per tissue type; iWAT Week 2-6: N = 5 mice per postnatal week). In **c, d**, each point represents the mean value per postnatal week (Week 2: N = 2 mice; Week 3-6: N = 3 mice). The error bars indicate mean

±*±*

SD.


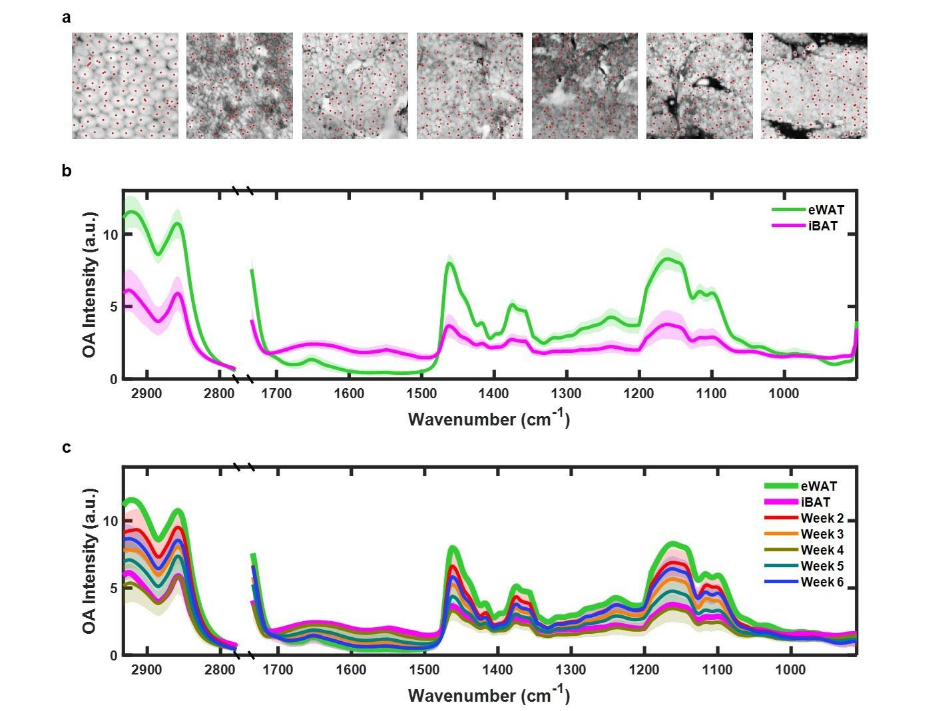


**Supplementary Figure S8. OA spectra of eWAT, iBAT, and iWAT of postnatal week 2-6. (a)** Representative micrographs at 2856 cm^-1^ of eWAT, IBAT, and iWAT of postnatal week 2-6 with randomly selected spectral acquired points (near the center of adipocytes) marked by red dots. **(b, c)** Depth-selective OA spectra of eWAT, iBAT, and iWAT of postnatal week 2-6 for the entire spectral range of QCL (2932-909 cm^-1^). The mean spectra (solid line) and their SD (shaded area) are shown for eWAT and iBAT (resulting spectra from 771 eWAT adipocytes and 745 iBAT adipocytes – 7 mice per tissue type), and iWAT (resulting spectrum from 525 adipocytes of Week 2, 525 adipocytes of Week 3, 537 adipocytes of Week 4, 535 adipocytes of Week 5, and 525 adipocytes of Week 6 – 5 mice per postnatal week).
